# Increased Cardiometabolic Risk in Men with Hypoprolactinemia: A Pilot Study

**DOI:** 10.3390/biom14101335

**Published:** 2024-10-20

**Authors:** Robert Krysiak, Karolina Kowalcze, Witold Szkróbka, Bogusław Okopień

**Affiliations:** 1 Department of Internal Medicine and Clinical Pharmacology, Medical University of Silesia, 40-752 Katowice, Poland; wszkrobka@sum.edu.pl (W.S.); bokopien@sum.edu.pl (B.O.); 2 Department of Pediatrics in Bytom, School of Health Sciences in Katowice, Medical University of Silesia, 41-902 Bytom, Poland; kkowalcze@sum.edu.pl; 3 Department of Pathophysiology, Faculty of Medicine, Academy of Silesia, Rolna 43, 40-555 Katowice, Poland

**Keywords:** cardiovascular risk factors, dopamine agonists, insulin sensitivity, prolactin deficiency

## Abstract

Low prolactin levels in men predispose them to mood disturbances, sexual dysfunction, and diabetes. The purpose of the current study was to assess cardiometabolic risk in males with hypoprolactinemia. This prospective study included three age-matched groups of young and middle-aged men: individuals with cabergoline-induced hypoprolactinemia (*n* = 15), cabergoline-treated subjects with prolactin levels within the reference range (*n* = 20), and untreated men with normal prolactin levels (*n* = 31). In men with hypoprolactinemia, the cabergoline dose was reduced in order to normalize prolactin concentration. Anthropometric parameters, blood pressure, QRISK3 score; plasma concentrations of prolactin, glucose, insulin, lipids, uric acid, high-sensitivity C-reactive protein (hsCRP), fibrinogen, homocysteine, and testosterone; whole-blood levels of glycated hemoglobin (HbA_1C_); urinary albumin-to-creatinine ratio (UACR); and carotid intima–media thickness were assessed at baseline and six months later. Men with hypoprolactinemia were characterized by higher body mass index, fat content, waist circumference, systolic blood pressure, fasting and 2 h post-load glucose, HbA_1C_, HOMA1-IR, uric acid, hsCRP, fibrinogen, homocysteine, and UACR; by lower HDL cholesterol and testosterone; by greater intima–media thickness; and by a higher QRISK3 score than their peers with normal prolactin levels. There were no statistically significant differences in the measured parameters between both groups of men with normal prolactin levels. Normalization of prolactin concentration was accompanied by normalization of biochemical variables, systolic blood pressure, and QRISK3 score. Although cabergoline dose reduction did not cause statistically significant changes in the remaining anthropometric parameters and intima–media thickness, six months later, they did not differ from those observed in the remaining study groups. Our findings suggest that iatrogenic hypoprolactinemia is associated with increased cardiometabolic risk, which is reversible and resolves after the normalization of prolactin levels.

## 1. Introduction

Until recently, hypoprolactinemia attracted little interest among endocrinologists, and it was assumed that the only clinical manifestation of low prolactin concentration is the absence of lactation after delivery [1]. Consequently, prolactin levels below 162 mU/L (7.6 ng/mL) at withdrawal are still regarded as the goal of long-term cabergoline treatment and, together with maximum tumor size below 3.1 mm, predict remission of prolactin-secreting tumors in the highest percentage of patients [2]. However, recent research provides increasing evidence that hypoprolactinemia cannot be regarded as a laboratory finding without relevant clinical consequences but should be considered a distinct clinical entity. Because changes in prolactin secretion play a role in the regulation of sexual responses and emotions, most studies concentrated on the association between prolactin deficiency and sexual functioning and/or mental disorders [3,4,5,6,7]. Low prolactin levels in men were characterized by reduced sexual desire, impaired erectile function, increased risk of premature ejaculation, and reduced overall sexual activity [3,4,5]. In turn, premenopausal women with prolactin levels below 5 ng/mL were characterized by abnormally low sexual desire and arousal [6]. Moreover, prolactin concentrations below 9.83 μg/L predicted hypoactive sexual desire disorder and a lower sexual inhibitory trait in both premenopausal and postmenopausal women [7]. Lastly, individuals with low prolactin levels more frequently met the criteria of anxiety [3] and depression [4,5,6].

The results of a few studies conducted to date suggest that prolactin deficiency may be also associated with metabolic or cardiovascular complications. The adjusted risk of developing type 2 diabetes was increased in European men aged between 40 and 80 years with prolactin concentration below 5 ng/mL and was particularly high in individuals with prolactin levels not exceeding 3 ng/mL [8]. However, the association between low prolactin and type 2 diabetes was less pronounced in men than in women [9]. Among men consulted for sexual dysfunction, those with prolactin levels in the lowest quartile were characterized by the highest risk of metabolic syndrome and lowest penile vascular flow [3]. Serum concentrations of prolactin below 12 ng/mL in both sexes were associated with adipocyte hypertrophy in visceral (but not in subcutaneous) adipose tissue, and with impaired insulin sensitivity compared to their peers with higher prolactin concentrations [10]. Iatrogenic hypoprolactinemia had a negative effect on the cardiometabolic profile of women of reproductive age [11]. In young women, prolactin deficiency also impaired insulin sensitivity and attenuated other cardiometabolic effects of atorvastatin [12]. Lastly, in addition to differences in fasting glucose and plasma lipids, women with hormone concentrations below 9.83 μg/L were characterized by higher values of body mass index (BMI), blood pressure (mainly systolic), and waist circumference, but they were younger than control women with prolactin concentrations exceeding this threshold [7]. Circulating prolactin levels were lower in individuals with ultrasound-diagnosed non-alcoholic fatty liver disease than in those without this complication and were lower in severe than mild-to-moderate liver steatosis [13]. The association with low prolactin production, not with the underlying condition, is supported by the finding that prolactin-receptor-null mice challenged with a high-fat diet developed greater insulin resistance, glucose intolerance, and increased adipocyte hypertrophy compared to wild-type mice [14]. Lastly, despite substitution with glucocorticoids, levothyroxine, and often also sex hormones and recombinant growth hormone, individuals with hypopituitarism were characterized by excess mortality, which was attributable mainly to cardiovascular and respiratory diseases [15].

While cardiometabolic risk in women with prolactin deficiency has been studied [11], no previous study was dedicated to investigating cardiometabolic risk in males with prolactin deficiency. To fill this gap, the aim of this study was to understand the association between low prolactin levels and risk factors for vascular and metabolic complications in men. Despite the multifactorial etiology of prolactin deficiency, most cases of hypoprolactinemia are secondary to treatment with dopamine agonists (mainly cabergoline) [1] or aripiprazole [16]. In order to minimize the impact of confounding factors, recruit enough patients, and obtain a homogeneous study population, this study included only men with hypoprolactinemia induced by cabergoline treatment.

## 2. Materials and Methods

This research was a prospective, matched, outpatient cohort study, performed in the periods between May 2018 and January 2020 and between January 2022 and May 2024 (participants were not recruited and followed up during the COVID-19 pandemic). All procedures were conducted in line with the Declaration of Helsinki, and the study protocol was approved by the institutional committee on human research. Written informed consent was obtained from all included participants after the required procedures and the details of this study were explained. No international registration was needed owing to the design of this study, which did not meet the criteria of a clinical trial. 

### 2.1. Study Population

**Figure 1:**
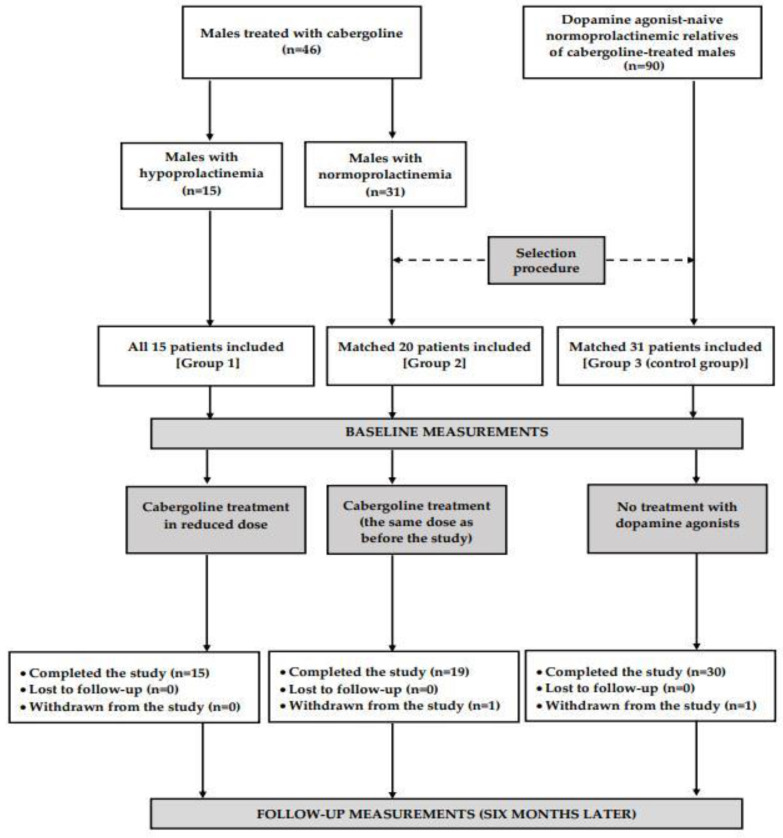
A diagram presenting the flow of patients in this study.

The participants of this study were recruited among men who had been initially supervised by local healthcare providers cooperating with our research group and were screened at our outpatient clinic for the presence of metabolic disorders. These individuals, aged from 20 to 65 years, were assigned to one of three study groups. Groups 1 and 2 had been receiving cabergoline for at least six months due to hyperprolactinemia secondary to traumatic brain injury or empty sella syndrome, idiopathic hyperprolactinemia, or idiopathic male infertility. Both groups differed in prolactin levels, assessed twice in the last three months. The minimum time period between both measurements was six weeks. Group 1 included 15 males with prolactin concentrations consistently below 3 ng/mL, while group 2 consisted of individuals with prolactin concentrations consistently within the reference range (between 3 and 20 ng/mL). The duration of hypoprolactinemia and normoprolactinemia was calculated based on the participants’ medical records. Group 3, which served as a control group, included 30 men untreated with dopaminergic agents or other drugs known to affect prolactin levels and/or glucose homeostasis. All prolactin measurements (including at least one during the last three months) in patients assigned to this group were required to be within normal limits. Group 3 was recruited among 90 relatives of cabergoline-treated individuals considered for enrollment in this study, and no subject was allowed to meet the exclusion criteria. Potential participants were excluded if prolactin levels were elevated (exceeded 20 ng/mL) or the results were inconsistent (e.g., one value suggestive of prolactin deficiency, with another suggesting normoprolactinemia). All men with hypoprolactinemia were enrolled, while the remaining groups were selected from a larger number of males meeting respective inclusion and exclusion criteria on the basis of the minimum Euclidean distance rule (Figure 1). The aim of this procedure was to obtain the study groups matched for age, smoking habits, and comorbidities, as well as to match groups 1 and 2 for weekly cabergoline dose and reasons for cabergoline treatment. In order to limit the impact of seasonal fluctuations in the outcome variables, 32 men (7 in group 1, 10 in group 2, and 15 in group 3) were recruited between December and February, and the remaining 34 between June and August. Moreover, to support or eliminate the impact of differences in fat content on the obtained results, we performed a post hoc comparison of 12 patients from each group with similar values of BMI.

The remaining exclusion criteria were as follows: prolactin-secreting tumors, mixed pituitary tumors, other hormonally active and non-functioning pituitary tumors, macroprolactinemia, galactorrhea, hypopituitarism, thyroid disorders (hypothyroidism, hyperthyroidism, or thyroid cancer), parathyroid disorders (hyperparathyroidism or hypoparathyroidism), diabetes, Cushing’s syndrome, Addison’s disease, congenital adrenal hyperplasia, aldosteronism, pheochromocytoma/paraganglioma, primary or secondary hypogonadism, neuroendocrine tumors, autoimmune diseases, renal or liver insufficiency, and other serious disorders. We also excluded men receiving drugs affecting prolactin levels, affecting glucose homeostasis, known to affect the remaining outcome measures, or interacting with dopamine agonists. 

### 2.2. Study Design

During the first visit, participants were asked to fill in a questionnaire evaluating how often and in what amount in the past two months they had consumed each of the most commonly used meals of Polish cuisine. Food intake frequency was assessed in terms of the following six categories: every day, 5–6 times per week, 3–4 times per week, 1–2 times per week, less than once per week, and never. Based on these data and Polish food-composition tables, the mean daily intake of proteins, lipids, carbohydrates, alcohol, and calories was calculated. In group 1, the dose of cabergoline was reduced in order to obtain prolactin concentrations within the reference range. In group 2, the drug was administered at the same doses as before the beginning of this study. In both groups, over the entire study period, the drug was administered orally once or twice a week at bedtime. Patients assigned to group 3 did not receive any dopaminergic agent. All participants were also instructed to comply with the goals of lifestyle modification over the entire study period. They were required to restrict intakes of total fat to less than 30% of energy intake, saturated fat to less than 7% of energy intake, and cholesterol to less than 200 mg per day and were encouraged to increase fiber intake to 15 g per 1000 kcal. The participants were also recommended to perform at least 150 min of moderate-intensity aerobic physical activity per week. The short-term use (for less than seven days) of new prescription or over-the-counter medications was permitted if the treatment was discontinued at least six weeks prior to the completion of this study. In patients receiving cabergoline because of iatrogenic hyperprolactinemia, antipsychotics were administered at the same dose as before this study. The withdrawal criteria included consent withdrawal, any exclusion criteria not detected upon recruitment, poor medication adherence, serious adverse effects, and other changes in pharmacological treatment. Serious adverse effects were defined as a life-threatening adverse event, inpatient hospitalization or prolongation of existing hospitalization (for > 24 h), a persistent or significant incapacity or substantial disruption of the ability to conduct normal life functions, an event jeopardizing the subject, or an event requiring medical or surgical intervention to prevent one of these outcomes [17]. Medication adherence was assessed every eight weeks by counting the number of unused tablets and was considered satisfactory if the percentage of tablets returned was in the range of 0% to 10%. Adherence to non-pharmacological recommendations was assessed by analysis of individual dietary questionnaires and diaries in which the participants recorded all their activities.

### 2.3. Measurements

Blood pressure was measured using a Riva Rocci sphygmomanometer on the nondominant arm at 5-minute intervals, starting 15 min after the patient had sat down. Korotkoff sounds 1 and 5 were used to define systolic and diastolic blood pressure, respectively. To eliminate possible confounding factors, blood pressure was measured three times, and the average of the available readings was used for analysis. BMI was defined as the body mass divided by the square of the body height, while fat-free mass index was calculated by dividing fat-free mass by height squared. Fat content was assessed using bioelectrical impedance analysis (model BF-511 B, Omron Healthcare Europe, Hoofddorp, the Netherlands), which is based on different conductance and impedance of fat and fat-free tissue. The waist circumference was measured at the midpoint between the lower border of the rib cage and the iliac crest by using a flexible tape. Common carotid intima–media thickness was measured by the same investigator (one of the coauthors) in the longitudinal plane, near (10 mm) the carotid bifurcation, as the distance from the intima–lumen interface to the media–adventitial border. Intima–media thickness was measured using high-resolution ultrasonography (Toshiba Aplio-500, Otarawa, Japan) on both sides of the neck, and the mean diameter was calculated. A person’s risk of developing myocardial infarction or stroke over the next 10 years was calculated using the QRISK 3-2018 Risk Calculator (https://qrisk.org). For men aged between 20 and 24 years at presentation, an age of 25 was assigned, as the QRISK 3-2018 Risk Calculator is only applicable to subjects aged 25 years or older. At the end of this study, all cabergoline-treated patients also underwent a standard echocardiographic examination. 

### 2.4. Laboratory Assays

All laboratory assays were performed on the first and last day of this study. Venous blood samples were collected from the antecubital vein between 7.00 and 8.00 a.m. after 12 h overnight fasting and assayed in duplicate to reduce intra-assay variation. Glucose levels were additionally measured in samples collected 2 h after the consumption of 75 g of glucose dissolved in 250 mL water. Plasma levels of glucose, total cholesterol, high-density lipoprotein (HDL) cholesterol, low-density lipoprotein (LDL) cholesterol, triglycerides, and uric acid and urine concentrations of albumin and creatinine were measured with standard methods using kits purchased from Roche Diagnostics (Roche Diagnostics, Basel, Switzerland). Glycated hemoglobin (HbA_1c_) was measured in whole-blood samples using a Cobas Integra 800 turbidimetric inhibition immunoassay (Roche Diagnostics, Mannheim, Germany). Plasma concentrations of prolactin, insulin, homocysteine, and testosterone were measured using acridinium ester-based direct chemiluminescence (ADVIA Centaur XP Immunoassay System, Siemens Healthcare Diagnostics, Munich, Germany). Plasma concentrations of high-sensitivity C-reactive protein (hsCRP) were measured by immunoassay with chemiluminescent detection (Immulite 2000XPi, Siemens Healthcare, Warsaw, Poland), while fibrinogen was assessed using the Clauss method, measuring the rate of fibrinogen to fibrin conversion in the presence of excess of thrombin (BCS XP autoanalyzer, Siemens Healthcare, Warsaw, Poland). Homeostasis model assessment 1 of insulin resistance ratio (HOMA1-IR), a marker of whole-body insulin sensitivity, was calculated by taking the product of the fasting values of glucose (in mg/dL) and insulin (in mU/L) and dividing it by 405 [18].

### 2.5. Statistical Analysis 

All variables were log-transformed to ensure that the data were normally distributed. Comparisons between the study groups were carried out using either analysis of variance followed by post hoc Bonferroni’s test (comparisons between three groups) or Student’s unpaired *t*-test (comparisons between two groups). Paired Student’s *t*-tests were used to compare the investigated variables within the same study group. The chi-square test was employed to compare qualitative variables. The relationships between the outcome variables were analyzed using Pearson’s correlation coefficient. To account for multiple comparisons, Bonferroni correction was applied, and adjusted two-sided *p*-values less than 0.05 were considered statistically significant. 

## 3. Results

Two patients prematurely terminated this study. One patient, assigned to group 2, poorly adhered to cabergoline treatment, which resulted in prolactin concentration exceeding the reference range. The second patient (from group 3) developed ulcerative colitis, which was one of the withdrawal criteria. The remaining 64 males (97%) completed this study, and their results were statistically analyzed. 

There were no statistically significant differences between the study groups in age, smoking habits, physical activity, education, occupational activity, type of work, stress exposure, percentage of patients with concomitant disorders, and percentage of patients receiving other drugs (Table 1 and Supplementary Table S1). There were no differences in indications for cabergoline treatment. Traumatic brain injury, empty sella syndrome, idiopathic hyperprolactinemia, and idiopathic male infertility were reported in six (40%), two (13%), three (20%), and four (27%) patients from group 1 and in seven (37%), three (16%), four (21%), and five (26%) patients from group 2 (*p* = 0.8018). The control group included 7 (23%) first-degree, 5 (17%) second-degree, 16 (53%) third-degree, and 2 (7%) fourth-degree relatives of cabergoline-treated individuals considered for enrollment in this study. The analyzed patients adhered to the treatment recommendations and to the recommendations concerning diet and physical activity and did not experience adverse effects associated with cabergoline treatment. The study groups did not differ in the mean daily intake of carbohydrates, lipids, cholesterol, proteins, alcohol, and calories, both before (Supplementary Table S2) and during this study (Supplementary Table S3). There were no cases of cardiac valvulopathy in cabergoline-treated patients.

At baseline, there were no differences between groups 1 and 2 in weekly and cumulative cabergoline dose, duration of cabergoline treatment, and duration of cabergoline-induced hypoprolactinemia or normoprolactinemia. Both groups were also comparable in terms of weekly cabergoline dose during this study and cumulative cabergoline dose at the end of this study (Table 2). Weekly cabergoline dose was higher at entry than during this study in group 1 (*p* = 0.0281) and was the same (no changes in dosage) in group 2 (*p* = 1.0000). 

At baseline, group 1 differed from the remaining groups in BMI, fat content, waist circumference, systolic blood pressure, common carotid intima–media thickness, and QRISK3 score, which were greater in group 1 than in the remaining two groups, but not in fat-free mass index and diastolic blood pressure. There were no differences in the anthropometric parameters, blood pressure, and intima–media thickness between groups 2 and 3. Cabergoline dose reduction led to a decrease in systolic blood pressure and QRISK3 score but did not affect BMI, free-fat mass index, fat content, waist circumference, diastolic blood pressure, and intima–media thickness. The assessed anthropometric parameters, blood pressure, intima–media thickness, and QRISK3 score remained at similar levels in groups 2 and 3. Six months after cabergoline dose reduction, there were no differences in all these variables between the study groups (Table 3).

Baseline prolactin concentration was lower in group 1 than in groups 2 and 3. Compared to patients with normal prolactin concentration, men with hypoprolactinemia had higher values of fasting and 2 h post-glucose load glucose, HbA_1C_, HOMA1-IR, uric acid, hsCRP, fibrinogen, homocysteine, and UACR and lower levels of HDL cholesterol and testosterone. There were no differences between group 1 and groups 2 and 3 in total cholesterol, LDL cholesterol, and triglycerides (Table 4). Differences in concentrations of 2 h post-load glucose, HbA_1C_, HOMA1-IR, HDL cholesterol, uric acid, hsCRP, fibrinogen, homocysteine, and UACR were also observed when subgroups with similar values of BMI were compared.

Cabergoline dose reduction was associated with an increase in prolactin; a decrease in glucose (both fasting and postchallenge), HbA_1C_, HOMA1-IR, uric acid, hsCRP, fibrinogen, homocysteine, and UACR; and an increase in HDL cholesterol and testosterone. Biochemical variables did not differ between groups 2 and 3, remaining at similar levels in these groups over the entire study period. Six months after cabergoline dose reduction, there were no between-group differences in these variables (Table 4).

**Figure 2:**
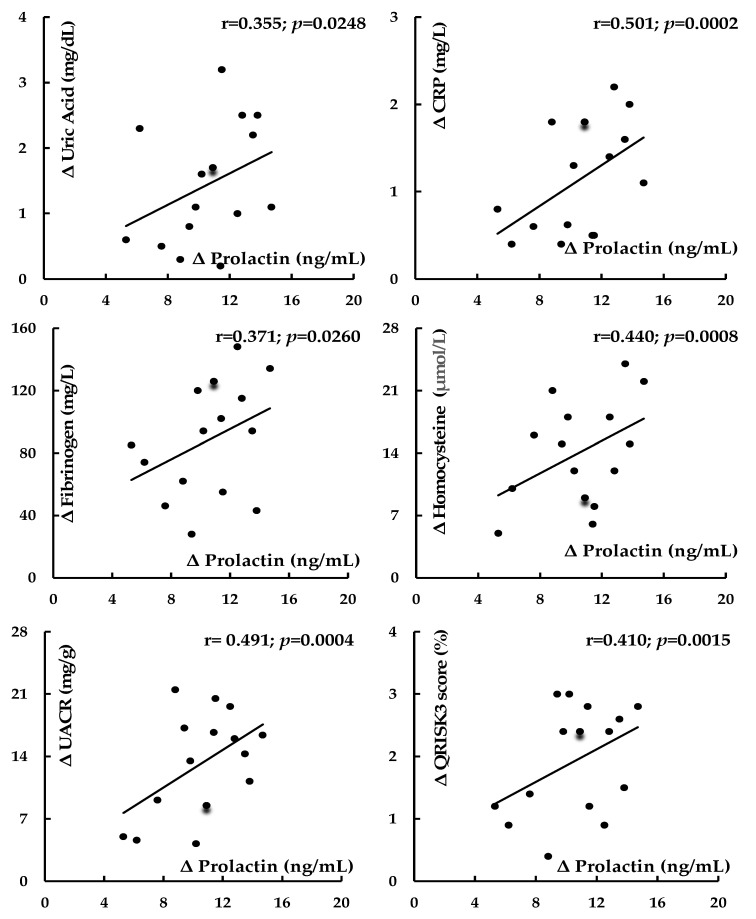
Correlations between cabergoline-dose-reduction-induced changes in prolactin levels and the changes in levels of cardiometabolic risk factors and QRISK3 score in in patients with iatrogenic hypoprolactinemia. Abbreviations: hsCRP—high-sensitivity C-reactive protein; UACR—urinary albumin-to-creatinine ratio.

In group 1, there were inverse correlations between prolactin concentration and BMI, fat content, waist circumference, systolic blood pressure, fasting glucose, 2 h post-load glucose, HOMA1-IR, HbA_1C_, uric acid, hsCRP, fibrinogen, homocysteine, UACR, intima–media thickness, and QRISK3 score and positive correlations between prolactin concentration and HDL cholesterol and testosterone. The cabergoline-dose-reduction-induced increase in prolactin levels correlated with the changes in systolic blood pressure, fasting glucose, 2 h post-load glucose, HOMA1-IR, HbA_1C_, HDL cholesterol, uric acid, hsCRP, fibrinogen, homocysteine, UACR, HDL cholesterol, testosterone, intima–media thickness, and QRISK3 score (Table 5 and Figure 2). There were no significant correlations between the investigated risk factors and the daily intake of energy and macronutrients (r values between −0.162 [*p* = 0.152] and 0.004 [*p* = 0.823]), between the investigated risk factors and testosterone levels (r values between −0.108 [*p* = 0.342] and 0.006 [*p* = 0.755]), and between the changes in the risk factors and the changes in BMI and fat content (r values between −0.006 [*p* = 0.755] and 0.149 [*p* = 0.236]). Other correlations were also insignificant.

## 4. Discussion

The major finding of the current study is that a low prolactin concentration in men is associated with increased cardiometabolic risk. This finding is in line with our previous observations concerning women [11]. Beyond sex, the participants of the present study were on average 15 years older than women participating in the previous one. Thus, prolactin deficiency seems to increase cardiometabolic risk in both sexes and in different age groups. The association between lactotroph hypofunction and the changes in the anthropometric parameters, systolic blood pressure, biomarkers, intima–media thickness, and risk of developing heart attack or stroke seems unequivocal. All variables that significantly differed between males with low and normal prolactin status correlated with the degree of prolactin deficiency. Moreover, there were positive correlations between the changes in these variables in response to cabergoline dose reduction and the increase in prolactin concentration. Owing to the matching procedure, our findings cannot be attributed to differences in age, smoking, comorbidities, and reasons for cabergoline treatment. Lastly, the study groups were comparable with respect to other aspects of sociodemographic characteristics and adherence to treatment recommendations.

Theoretically, higher BMI and fat content in patients with hypoprolactinemia compared to subjects with normal prolactin levels might have been secondary to the worst lifestyle behaviors in the former group. Although this explanation cannot be completely ruled out, it seems rather unlikely. The mean baseline daily intake of proteins, lipids, carbohydrates, alcohol, and calories and physical activity did not differ between the groups. Moreover, intake of energy and macronutrients did not change significantly during this study and did not correlate with the changes in the assessed cardiometabolic parameters in response to cabergoline dose reduction. Thus, the obtained results seem to support previous studies suggesting that hypoprolactinemia is intrinsically associated with increased fat content [7,10,14]. Interestingly, most differences in concentrations of cardiometabolic risk factors were observed even if we compared subgroups of hypoprolactinemic and normoprolactinemic patients with similar values of BMI, despite there being only 12 patients in each subgroup. This finding and the lack of correlations between the changes in BMI and in other outcome variables indicate that increased cardiometabolic risk in individuals with hypoprolactinemia cannot be regarded only as a consequence of higher fat content.

Although hypoprolactinemia was secondary to inadequately high doses of cabergoline in all patients, our findings cannot be explained by the impact of this agent. The doses of cabergoline (both weekly and cumulative) and treatment duration did not differ significantly between both groups of patients receiving this agent. Moreover, there were no differences in the anthropometric parameters, QRISK3 score, blood pressure, biochemical variables assessed in our study, and intima–media thickness between individuals not receiving dopaminergic agents and normoprolactinemic men treated with cabergoline owing to previously diagnosed prolactin excess. Furthermore, at entry, the assessed variables did not correlate with cabergoline dose and treatment duration. Lastly, there were no correlations between the improvement in the cardiometabolic profile and the degree of reduction in cabergoline dose. The lack of association between increased cardiometabolic risk in iatrogenic hypoprolactinemia and cabergoline treatment is supported by observations of individuals with prolactin excess. Treatment of patients with elevated levels of this hormone with dopamine agonists lowered BMI, waist circumference, visceral adiposity, glucose, and HbA_1C_; increased insulin sensitivity; and improved plasma lipids [19,20,21,22,23,24]. Dopaminergic agents were also found to decrease blood pressure, reduce systemic inflammation, improve flow-mediated dilatation, and decrease intima–media thickness [25,26,27]. These cardiometabolic effects were more pronounced in individuals receiving cabergoline than bromocriptine [28]. Lastly, patients with prolactinoma who relapsed after cabergoline treatment were characterized by higher fibrinogen, lower HDL cholesterol, and greater intima–media thickness than patients entering remission [29]. All these findings consistently indicate that increased cardiometabolic risk in our study was associated with low prolactin status.

It should be underlined that despite treatment with inappropriately high doses of cabergoline, there were no cases of cardiac valve disease in the study population. This complication, attributed to its affinity for the serotonin receptor subtype 2B, abundantly expressed in heart valves, which mediate mitogenesis, fibroblast proliferation, and remodeling, was reported mainly in patients with Parkinson’s disease chronically treated with very high cumulative doses of cabergoline (2600–6700 mg) [30,31]. In patients with prolactin excess, who usually receive much lower doses of cabergoline, this risk is negligible, even if treatment is long-term [30,32]. Our observations are in line with this opinion. Despite chronic cabergoline administration, the cumulative cabergoline dose at the end of this study was relatively low (73.6 and 71.6 mg [depending on the group]). Thus, iatrogenic hypoprolactinemia may pose a more serious health threat for cabergoline-treated young or middle-aged men with non-tumoral prolactin excess than valvulopathy. However, this question should be verified in future studies. Relatively low doses of cabergoline may also explain why no patient developed impulse control disorders, such as gambling, compulsive shopping, binge eating, and hypersexuality (so-called “dopa-testotoxicosis”) [33,34]).

Another interesting finding was that men with hormone levels below 3 ng/mL were characterized by a greater thickness of the intima–media complex, which is a reliable marker of early atherosclerosis and an independent risk factor for vascular events and cardiovascular mortality [35,36]. This finding, which is in line with previous observations concerning young women [11], indicates that hypoprolactinemia may predispose adults to asymptomatic atherosclerosis, which may develop in premenopausal women and young and middle-aged men. It should be stressed that statistically significant differences in this parameter were observed despite the fact that the average treatment duration in hypoprolactinemic men was only 10 months, while the average period of prolactin deficiency was even shorter (7 months). Thus, atherosclerotic changes in the vascular wall may appear shortly after the onset of this abnormality.

The study population was characterized by the same etiology of prolactin deficiency. Although iatrogenic hypoprolactinemia is by far the most common reason for this disorder, low prolactin levels may be also secondary to genetically determined defects of its production or may be one of the manifestations of other pituitary disorders, including pituitary tumors, infiltrative diseases, hypophysitis, extensive pituitary surgery, massive cerebral trauma, pituitary ischemia or hemorrhage, high-dose radiotherapy, or chemotherapy [1]. Considering that some coexisting disorders, such as cortisol deficiency, hypogonadism, and growth hormone deficiency, increase cardiometabolic risk [37,38,39], this risk may be greater if the underlying disorder impairs the function of not only lactotrophs but also other types of pituitary cells. Our previous [11] and present findings may partially explain why individuals with hypopituitarism are at higher risk of death even if other deficient hormones are replaced [15]. Unfortunately, drugs increasing prolactin levels in patients without pituitary disorders, such as metoclopramide, domperidone, and antipsychotics, not only cause adverse effects but were not found to increase plasma hormone levels in patients with prolactin deficiency of organic origin [1,40]. Theoretically, such patients, at least those at high cardiometabolic risk, should be candidates for treatment with recombinant prolactin. Unfortunately, to the best of our knowledge, no human or animal study has investigated the impact of prolactin substitution on circulating prolactin levels and other markers of prolactin deficiency, and therefore, its safety and effectiveness have not been proven. However, these patients may benefit from insulin-sensitizing, hypolipemic, or hypotensive agents that have additional pleiotropic effects, including metformin, 3-hydroxymethyl-3-glutaryl coenzyme A reductase inhibitors, angiotensin-converting enzyme inhibitors, and angiotensin receptor blockers.

The obtained results also allow us to draw other conclusions. Firstly, considering that increased cardiometabolic risk is a characteristic feature of prolactin excess [19,20,21,22,23,24,25,26,27], our findings provide arguments that the association between prolactin levels and cardiometabolic complications may be U-shaped. Thus, the treatment of prolactin excess should be directed at avoiding both persistent lactotroph hyperfunction and lactotroph failure. Secondly, no baseline differences between patients with cabergoline-induced normoprolactinemia and those belonging to the control group exclude that our findings might have resulted from a persistent effect of prolactin excess, which prompted treatment with cabergoline. Thirdly, we reported 15 men with cabergoline-induced hypoprolactinemia in one research center, though they were treated with relatively low doses of this drug. This indicates that prolactin deficiency (at least iatrogenic), though infrequent, is probably underreported. Therefore, it seems that prolactin concentration should be periodically assessed in all patients receiving this agent and probably also other drugs found to induce lactotroph hypofunction. Last but not least, increased cardiometabolic risk in men with cabergoline-induced hypoprolactinemia is reversible and resolves after dose reduction. In line with this explanation, at the end of the study period, there were no between-group differences in the outcome measures. Moreover, cabergoline dose reduction led to statistically significant changes in all measured biochemical variables, systolic blood pressure, and overall cardiovascular risk, the degree of which correlated with the increase in prolactin levels. The lack of statistically significant differences between baseline and follow-up BMI, fat content, waist circumference, and intima–media thickness in individuals with iatrogenic hypoprolactinemia may be explained by the fact that they represent structural changes in adipose tissue size and distribution, and in the vascular wall, and therefore require more time to be noticeable than the changes in biomarkers.

We previously reported that the unfavorable effect of hypoprolactinemia on desire and erectile function was partially mediated by a decrease in testosterone concentration [4]. Moreover, an association between lactotroph hypofunction and testicular failure in men was found by other researchers and was attributed to impaired gonadotropin receptor action at the testicular level [41,42]. In the current study, we also observed lower testosterone levels in prolactin-deficient men. The association between decreased testosterone production and the low prolactin status of patients at baseline and in response to treatment modification indicates that mildly reduced testosterone concentration was secondary to prolactin deficiency. However, the lack of correlations with the anthropometric variables, systolic blood pressure, biomarkers, intima–media thickness, and QRISK3 score before cabergoline dose reduction and six months later strongly argues against the role of low testosterone as a mediator of cardiovascular and metabolic complications of prolactin deficiency. Thus, functional changes in testicular function likely mediate the impact of prolactin deficiency on various aspects of sexual health but do not seem to contribute to increased cardiometabolic risk in males with this disorder. A much more likely explanation is that cardiometabolic effects result from inadequate stimulation of the prolactin receptor in the target cells, mediating the action of prolactin on pancreatic islet cell function, fat store distribution, glucose and lipid metabolism, and energy homeostasis [43]. Prolactin deficiency may also have an impact on monoaminergic pathways in the hypothalamus and in other brain regions, modulated by this hormone and playing a role in the central regulation of blood pressure, glucose homeostasis, lipid production and metabolism, and systemic inflammation [44,45].

There are several shortcomings of this study that warrant caution in interpreting the results. Hypoprolactinemia is very rarely diagnosed and asymptomatic in most males [1], which limited the number of participants. Thus, our single-center study should be regarded as a pilot one, and the obtained results need to be confirmed in a multi-center study with a larger sample size. There is no universally accepted cut-off value allowing the diagnosis of prolactin deficiency. The chosen value (3 ng/mL) was a threshold concentration below which the risk of type 2 diabetes in men was highest [8]. Theoretically, the obtained results might have been different if another cut-off value had been chosen. This study assessed only surrogate outcomes but not clinically relevant endpoints (cardiovascular and metabolic morbidity and mortality). Owing to the enrollment of relatives as controls, we cannot completely exclude the possibility that genetic factors might have influenced our findings. Because of the chosen study design, the results might have also been affected by other confounding factors and selection bias. Lastly, precautions during study design and data analysis limited, but did not completely eliminate, the regression toward the mean [46]. 

## 5. Conclusions

Low prolactin levels in men receiving even moderate cabergoline doses were accompanied by unfavorable changes in their body mass, fat distribution, blood pressure, vascular wall, and calculated risk of cardiovascular events and by higher plasma and urine concentrations of cardiometabolic risk factors compared to individuals with prolactin concentrations within the reference range. Differences between patients with low and normal prolactin status were not observed six months after the normalization of prolactin levels. As the major novelty and strength of this study, the obtained results suggest that iatrogenic hypoprolactinemia increases cardiometabolic risk in men and that its effective treatment may eliminate this risk. Further research is needed to investigate whether individuals with organic prolactin deficiency benefit from substitution with exogenous prolactin.

## Figures and Tables

**Table 1:** Sociodemographic characteristics of the study population.

Variable	Group 1	Group 2	Group 3	*p*-Value		
				1 vs. 2	1 vs. 3	2 vs. 3
Number of patients	15	19	30	-	-	-
Age (years)	46 ± 13	47 ± 12	48 ± 14	0.8176	0.6462	0.7983
Smokers (*n* (%)) Number of cigarettes a day (*n*) Duration of smoking (years)	6 (40) 11 ± 7 24 ± 10	7 (37) 12 ± 8 26 ± 11	12 (40) 10 ± 9 26 ± 12	0.8495 0.8156 0.7398	1.0000 0.8154 0.7295	0.8248 0.6445 1.0000
Physical activity: total/once a week/several times a week/once a month (%)	94/27/40/27	89/26/42/21	94/27/47/20	0.8769	0.7885	0.8038
Primary or vocational/secondary/university education (%)	20/40/40	21/37/42	7/40/43	0.8674	0.6534	0.6781
Occupational activity/blue-collar/white-collar/pink-collar workers (%)	94/47/47/0	100/42/53/5	97/43/47/7	0.8224	0.8628	0.8815
Stress exposure (*n* (%))	11 (73)	15 (79)	23 (76)	0.7012	0.8062	0.8521
Concomitant disorders (*n* (%))	3 (20)	4 (21)	7 (23)	0.9325	0.7988	0.8516
Comedications (*n* (%))	2 (13)	3 (16)	5 (17)	0.8415	0.7706	0.9383

Unless otherwise stated, the data are presented as the mean ± standard deviation. Stress exposure was assessed using contextual threat-based interview methods and defined as exposure to at least one chronic stressor. Group 1: men with cabergoline-induced hypoprolactinemia. Group 2: men with prolactin levels within the reference range treated with cabergoline. Group 3: dopamine-agonist-naïve men with prolactin levels within the reference range.

**Table 2:** Details of cabergoline treatment and duration of hypoprolactinemia/normoprolactinemia in cabergoline-treated patients.

Variable	Group 1 (*n* = 15)	Group 2 (*n* = 19)	*p*-Value (1 vs. 2)
Cabergoline dose before this study (mg weekly)	1.28 ± 0.51	1.09 ± 0.40	0.2318
Cumulative cabergoline dose before this study (mg)	49.8 ± 11.3	43.4 ± 9.5	0.0822
Duration of cabergoline treatment before this study (months)	10 ± 2	9 ± 2	0.1818
Duration of hypoprolactinemia/normoprolactinemia before this study (months)	7 ± 2	6 ± 2	0.1785
Cabergoline dose during this study (mg weekly)	0.92 ± 0.32	1.09 ± 0.40	0.1060
Cumulative cabergoline dose at the end of this study (mg)	73.6 ± 16.4	71.6 ± 14.8	0.7115

The data are presented as the mean ± standard deviation. Group 1: men with cabergoline-induced hypoprolactinemia. Group 2: men with prolactin levels within the reference range treated with cabergoline.

**Table 3:** Anthropometric parameters, blood pressure, intima–media thickness, and 10-year risk of developing myocardial infarction or stroke in the study population.

	Group 1	Group 2	Group 3
BMI (kg/m^2^)			
At baseline	27.0 ± 4.8 ^a^	23.8 ± 4.2	23.4 ± 4.4
After 6 months	25.3 ± 4.0	24.2 ± 4.1	23.5 ± 3.8
Fat-free mass index (kg/m^2^)			
At baseline	19.5 ± 3.4	19.0 ± 3.0	18.9 ± 2.8
After 6 months	19.4± 2.9	19.1 ± 3.2	18.7 ± 3.8
Fat content (%)			
At baseline	25.1 ± 6.0 ^a^	20.3 ± 5.5	19.8 ± 4.9
After 6 months	23.8 ± 5.8	21.2 ± 5.0	20.8 ± 5.3
Waist circumference (cm)			
At baseline	101 ± 12 ^a^	92 ± 10	91 ± 15
After 6 months	97 ± 15	93 ± 13	92 ± 16
Systolic blood pressure (mmHg)			
At baseline	134 ± 18 ^a^	123 ± 12	118 ± 14
After 6 months	123 ± 16 ^b^	119 ± 20	117 ± 15
Diastolic blood pressure (mmHg)			
At baseline	82 ± 10	80 ± 6	79 ± 6
After 6 months	80 ± 9	81 ± 7	79 ± 8
Carotid intima–media thickness (mm)			
At baseline	0.81 ± 0.10 ^a^	0.71 ± 0.07	0.70 ± 0.06
After 6 months	0.75 ± 0.09	0.72 ± 0.07	0.71 ± 0.06
QRISK3 score (%)			
At baseline	5.6 ± 1.9 ^a^	3.7 ± 1.3	3.4 ± 1.2
After 6 months	3.7 ± 1.2 ^b^	3.5 ± 1.4	3.4 ± 1.1

The data are presented as the mean ± standard deviation. Ten-year risk of developing myocardial infarction or stroke was calculated using the QRISK-3-2018 risk calculator. ^a^ *p* < 0.05 vs. groups 2 and 3; ^b^ *p* < 0.05 vs. baseline value. Group 1: men with cabergoline-induced hypoprolactinemia. Group 2: men with prolactin levels within the reference range treated with cabergoline. Group 3: dopamine-agonist-naïve men with prolactin levels within the reference range. Abbreviation: BMI—body mass index.

**Table 4:** Biochemical variables in the study population.

Variable	Group 1	Group 2	Group 3
Prolactin (ng/mL)			
At baseline	1.8 ± 0.8 ^a^	12.5 ± 4.6	12.9 ± 4.0
After 6 months	12.4 ± 4.1 ^b^	12.8 ± 4.6	13.1 ± 4.2
Fasting glucose (mg/dL)			
At baseline	97 ± 12 ^a^	87 ± 9	85 ± 10
After 6 months	88 ± 10 ^b^	86 ± 8	84 ± 7
2 h post-load glucose (mg/dL)			
At baseline	138 ± 18 ^a^	120 ± 15	116 ± 12
After 6 months	123 ± 20 ^b^	118 ± 17	118 ± 18
HbA1C (%)			
At baseline	5.8 ± 0.5 ^a^	5.2 ± 0.4	5.4 ± 0.4
After 6 months	5.4 ± 0.5 ^b^	5.3 ± 0.5	5.3 ± 0.5
HOMA1-IR			
At baseline	2.2 ± 0.8 ^a^	1.5 ± 0.4	1.4 ± 0.5
After 6 months	1.6 ± 0.6 ^b^	1.5 ± 0.6	1.5 ± 0.4
Total cholesterol (mg/dL)			
At baseline	193 ± 37	190 ± 31	182 ± 28
After 6 months	192 ± 40	188 ± 29	185 ± 29
HDL cholesterol (mg/dL)			
At baseline	39 ± 10 ^a^	52 ± 8	55 ± 9
After 6 months	51 ± 12 ^b^	55 ± 11	56 ± 10
LDL cholesterol (mg/dL)			
At baseline	118 ± 32	108 ± 22	98 ± 18
After 6 months	110 ± 29	104 ± 19	100 ± 16
Triglycerides (mg/dL)			
At baseline	168 ± 70	142 ± 63	132 ± 40
After 6 months	144 ± 58	130 ± 50	135 ± 48
Uric acid (mg/dL)			
At baseline	5.5 ± 1.6 ^a^	4.0 ± 1.4	4.2 ± 1.2
After 6 months	4.1 ± 1.8 ^b^	4.3 ± 1.6	3.9 ± 1.4
hsCRP (mg/L)			
At baseline	3.1 ± 1.2 ^a^	2.0 ± 0.8	1.9 ± 0.7
After 6 months	2.0 ± 0.9 ^b^	1.8 ± 0.7	1.7 ± 0.8
Fibrinogen (mg/dL)			
At baseline	408 ± 120 ^a^	305 ± 80	324 ± 69
After 6 months	320 ± 93 ^b^	295 ± 81	310 ± 76
Homocysteine (μmol/L)			
At baseline	34 ± 15 ^a^	17 ± 10	14 ± 8
After 6 months	20 ± 12 ^b^	18 ± 12	16 ± 9
UACR (mg/g)			
At baseline	32.8 ± 14.6 ^a^	16.3 ± 12.6	12.6 ± 10.5
After 6 months	19.5 ± 12.5 ^b^	15.1 ± 12.3	14.9 ± 11.3
Testosterone (ng/mL)			
At baseline	4.8 ± 1.3 ^a^	6.9 ± 2.5	7.1 ± 3.0
After 6 months	6.9 ± 2.1 ^b^	6.7 ± 2.8	7.3 ± 2.6

The data are presented as the mean ± standard deviation. ^a^ *p* < 0.05 vs. groups 2 and 3; ^b^ *p* < 0.05 vs. baseline value. Group 1: men with cabergoline-induced hypoprolactinemia. Group 2: men with prolactin levels within the reference range treated with cabergoline. Group 3: dopamine-agonist-naïve men with prolactin levels within the reference range. Abbreviations: HbA_1C_—glycated hemoglobin; HDL—high-density lipoprotein; HOMA1-IR—the homeostatic model assessment 1 of insulin resistance ratio; hsCRP—high-sensitivity C-reactive protein; LDL—low-density lipoprotein; UACR—urinary albumin-to-creatinine ratio.

**Table 5:** Correlations between the assessed variables in patients with cabergoline-induced hypoprolactinemia (Group 1).

Correlated Variables		Correlations at Baseline		Correlations Between Changes in Response to Cabergoline Dose Reduction	
		r Value	*p*-Value	r Value	*p*-Value
Prolactin	BMI	−0.328	0.0400	0.234	0.0688
Prolactin	Fat content	−0.411	0.0026	0.242	0.0556
Prolactin	Waist circumference	−0.352	0.0294	0.246	0.0510
Prolactin	Systolic blood pressure	−0.428	0.0019	0.435	0.0010
Prolactin	Fasting glucose	−0.302	0.0481	0.351	0.0254
Prolactin	2 h post-load glucose	−0.406	0.0016	0.395	0.0023
Prolactin	HOMA1-IR	−0.423	0.0012	0.406	0.0018
Prolactin	HbA_1C_	−0.345	0.0298	0.321	0.0418
Prolactin	HDL cholesterol	−0.362	0.0208	0.310	0.0480
Prolactin	Uric acid	−0.386	0.0065	0.355	0.0248
Prolactin	hsCRP	−0.468	0.0008	0.501	0.0002
Prolactin	Fibrinogen	−0.326	0.0406	0.371	0.0260
Prolactin	Homocysteine	−0.419	0.0014	0.440	0.0008
Prolactin	UACR	−0.456	0.0010	0.491	0.0004
Prolactin	Testosterone	0.482	0.0004	0.465	0.0004
Prolactin	Intima–media thickness	−0.388	0.0062	0.460	0.0005
Prolactin	QRISK3 score	−0.402	0.0018	0.410	0.0015

Abbreviations: BMI—body mass index; HbA_1C_—glycated hemoglobin; HDL—high-density lipoprotein; HOMA1-IR—the homeostatic model assessment 1 of insulin resistance ratio; hsCRP—high-sensitivity C-reactive protein; LDL—low-density lipoprotein; UACR—urinary albumin-to-creatinine ratio.

## Data Availability

The data that support the findings of this study are available from the corresponding author upon reasonable request.

## Supplementary Materials

The following supporting information can be downloaded at: https://www.mdpi.com/article/10.3390/biom14101335/s1. Table S1: Comorbidities and comedications in the study population; Table S2: Mean daily nutrient and calorie intake in the study population during the last two months before this study; Table S3: Mean daily nutrient and calorie intake in the study population during this study.

## Abbreviations

BMI	body mass index
HbA_1C_	glycated hemoglobin
HDL	high-density lipoprotein
HOMA1-IR	homeostasis model assessment 1 of insulin resistance ratio
LDL	low-density lipoprotein
UACR	urinary albumin-to-creatinine ratio
