# Supplementary material for: Increased Cardiometabolic Risk in Men with Hypoprolactinemia: A Pilot Study
Source: Biomolecules. 2024 Oct 20;14(10):1335. doi: 10.3390/biom14101335 (PMC11506548; doi:10.3390/biom14101335)
Supplement: Supplementary file 1 [file biomolecules-14-01335-s001.zip › biomolecules-3210757-supplementary.pdf]

biomolecules

MDPI

Supplementary materials

Increased Cardiometabolic Risk in Men with Hypoprolactinemia: A Pilot Study

Robert Krysiak [1,*], Karolina Kowalcze [2,3], Witold Szkróbka [1] and Bogusław Okopień [1]

[1] Department of Internal Medicine and Clinical Pharmacology, Medical University of Silesia, 40-752 Katowice, Poland; wszkrobka@sum.edu.pl (W.S.); bokopien@sum.edu.pl (B.O.)
[2] Department of Pediatrics in Bytom, School of Health Sciences in Katowice, Medical University of Silesia, 41-902 Bytom, Poland; kkowalcze@sum.edu.pl
[3] Department of Pathophysiology, Faculty of Medicine, Academy of Silesia, Rolna 43, 40-555 Katowice, Poland
* Correspondence: rkrysiak@sum.edu.pl

Table S1. Comorbidities and comedications in the study population.

	Group 1	Group 2	Group 3
Comorbidities	Hay fever (*n* = 1)<br>Cholelithiasis (*n* = 1)<br>Diverticulosis (*n* = 1)	Glaucoma (1)<br>Benign prostate hyperplasia (1)<br>Irritable bowel syndrome (*n* = 1)<br>Kidney cysts (1)	Panic disorder (*n* = 1)<br>Irritable bowel syndrome (*n* = 1)<br>Chronic urticaria (1)<br>Benign prostate hyperplasia (1)<br>Iron-deficiency anemia due to impaired iron absorption (1)<br>Varicocele (1)<br>Emphysema (1)
Comedications	Loratadine [antihistamine drug] (*n* = 1)<br>Drotaverine [antispasmodic drug] (*n* = 1)	Latanoprost [antiglaucoma drug] (*n* = 1)<br>Tamsulosin [uroselective α_{1A}/α_{1D} receptor antagonist] (*n* = 1)<br>Mebeverine [anticholinergic agent] (*n* = 1)	Alprazolam [benzodiazepine] (*n* = 1)<br>Mebeverine [anticholinergic agent] (*n* = 1)<br>Cetirizine [antihistamine drug] (*n* = 1)<br>Tamsulosin [uroselective α_{1A}/α_{1D} receptor antagonist] (*n* = 1)<br>Iron [parenteral supplementation] (*n* = 1)

Group 1: men with cabergoline-induced hypoprolactinemia. Group 2: men with prolactin levels within the reference range treated with cabergoline. Group 3: dopamine agonist-naïve men with prolactin levels within the reference range.

Table S2. Mean daily nutrient and calorie intake in the study population during the last two months before the study.

Nutrient/calorie intake	Group 1	Group 2	Group 3	*p*-value		
				1 *vs.* 2	1 *vs.* 3	2 *vs.* 3
Carbohydrate intake (g)	361 ± 95	352 ± 92	340±88	0.7819	0.4663	0.6731
Lipid intake (g)	73 ± 20	70 ± 18	78 ± 23	0.6490	0.4776	0.2049
Cholesterol intake (mg)	305 ± 90	289 ± 85	300 ± 78	0.5990	0.8482	0.6440
Protein intake (g)	68 ± 14	65 ± 18	62 ± 15	0.5994	0.2039	0.5311
Alcohol intake	12 ± 6	13 ± 7	14 ± 7	0.6630	0.3498	0.6284
Total calorie intake (kcal)	2529 ± 320	2446 ± 298	2476 ± 342	0.4407	0.6194	0.7549

Group 1: men with cabergoline-induced hypoprolactinemia. Group 2: men with prolactin levels within the reference range treated with cabergoline. Group 3: dopamine agonist-naïve men with prolactin levels within the reference range.

Table S3. Mean daily nutrient and calorie intake in the study population during the study.

Nutrient/calorie intake	Group 1	Group 2	Group 3	*p*-value		
				1 *vs.* 2	1 *vs.* 3	2 *vs.* 3
Carbohydrate intake (g)	362 ± 102	355 ± 97	348 ± 95	0.8394	0.6515	0.8005
Lipid intake (g)	63 ± 17	60 ± 15	64 ± 18	0.5888	0.8589	0.4240
Cholesterol intake (mg)	175 ± 28	168 ± 30	173 ± 26	0.4918	0.8137	0.5396
Protein intake (g)	75 ± 14	74 ± 18	72 ± 15	0.8607	0.5216	0.6953
Alcohol intake	11 ± 6	12 ± 6	12 ± 7	0.6327	0.6389	1.0000
Total calorie intake (kcal)	2462 ± 355	2429 ± 292	2398 ± 341	0.7690	0.5612	0.7450

Group 1: men with cabergoline-induced hypoprolactinemia. Group 2: men with prolactin levels within the reference range treated with cabergoline. Group 3: dopamine agonist-naïve men with prolactin levels within the reference range.
